# Real-time selective visual monitoring of Hg^2+^ detection at ppt level: An approach to lighting electrospun nanofibers using gold nanoclusters

**DOI:** 10.1038/srep10403

**Published:** 2015-05-28

**Authors:** Anitha Senthamizhan, Asli Celebioglu, Tamer Uyar

**Affiliations:** 1 UNAM-National Nanotechnology Research Center, Bilkent University, Ankara, 06800, Turkey; 2 Institute of Materials Science & Nanotechnology, Bilkent University, Ankara, 06800, Turkey

## Abstract

In this work, fluorescent gold nanocluster (AuNC) decorated polycaprolactone (PCL) nanofibers (AuNC*PCL-NF) for real time visual monitoring of Hg^2+^ detection at ppt level in water is demonstrated. The resultant AuNC*PCL-NF exhibiting remarkable stability more than four months at ambient environment and facilitates increased accessibility to active sites resulting in improved sensing performance with rapid response time. The fluorescence changes of AuNC*PCL-NF and their corresponding time dependent spectra, upon introduction of Hg^2+^, led to the visual identification of the sensor performance. It is observed that the effective removal of excess ligand (bovine serum albumin (BSA) greatly enhances the surface exposure of AuNC and therefore their selective sensing performance is achieved over competent metal ions such as Cu^2+^, Ni^2+^, Mn^2+^, Zn^2+^, Cd^2+^, and Pb^2+^ present in the water. An exceptional interaction is observed between AuNC and Hg^2+^, wherein the absence of excess interrupting ligand makes AuNC more selective towards Hg^2+^. The underlying mechanism is found to be due to the formation of Au-Hg amalgam, which was further investigated with XPS, TEM and elemental mapping studies. In short, our findings may lead to develop very efficient fluorescent-based nanofibrous mercury sensor, keeping in view of its stability, simplicity, reproducibility, and low cost.

Mercury contamination in the environment has adversely affected undoubtedly the water quality and continues to threaten the public health which is expected to be more aggravated in the future^1^. Various detection techniques have been devised, of which, fluorescent-based sensors have been identified as an efficient sensing platform with regard to their simple visual detection of metal ions in the environmental samples^2^,^3^,^4^,^5^,^6^,^7^,^8^,^9^,^10^. However, there occurs an immediate need and demand for miniaturizing the sensor to a single nanoprobe level that possesses the advantages of lower monetary benefits, high sensitivity with lower detection limit and a faster response time. Furthermore, lack of effective means to locate a specific probe using optical microscopy method following the correlation of their properties needs further research and study^11^. This represents an obstacle for studying the sensing response of the analyte with respect to an individual probe.

Because of the striking sensing performance of the fluorescent nanoparticles, substantial effort has already been made^12^,^13^,^14^,^15^. Nevertheless, nearly all the reported approaches have been found to be carried out in the solution state. Unfortunately, issues have been reported that solution-based active probes occasionally exhibit low fluorescence efficiency due to self-quenching caused by the environmental changes. Also, the solution-based detection technique greatly limits their usage in outdoor applications, owing to limited stability, material utilization and detection cost.

The method of attaching the probes on to a solid substrate is hopeful to prove desirable and an efficient, reliable and stable sensing platform^16^,^17^,^18^,^19^. However, the formation and application of such a hybrid solid platform for the sensing of metal ions is still in its infancy stage. Interestingly, a few studies have closely considered the usage of a solid support for development of sensor till date, though any major turnover is yet to arrive^20^,^21^,^22^,^23^,^24^. Also, in most of the cases, problems occurred owing to the contamination of the sample and the efforts required to curbing it. To circumvent the drawbacks mentioned above, we present here an environmental friendly hybrid system for the effective detection of Hg^2+^ in water.

The unique characteristics of electrospun nanofibers (NF) prove advantageous to meet the requirements of solid substrates and are likely to facilitate more active probes to analytes, thereby improving the sensitivity and response time in sensing applications^25^,^26^,^27^,^28^,^29^,^30^,^31^. By utilizing polycaprolactone (PCL) electrospun NF as a support matrix, it proves to be favorable to assemble the gold nanoclusters (AuNC) onto the NF, successfully minimizing undesirable nanoparticle aggregation, unlike in the other solid support. In view of increasing study and research on the applications of fluorescent AuNC for metal ion sensing makes the whole process more attractive and inspiring to use an active probe to make an environmental-friendly fluorescent sensor. Moreover, PCL is biodegradable; insoluble in water; mechanically strong and most importantly, biocompatible and highly efficient in both *in vitro* and *in vivo.* This has played a vital role in obtaining the approval of a number of PCL-based medical and drug-delivery devices by the U.S. Food and Drug Administration (FDA)^32^.

Recently, we have reported AuNC incorporated flexible electrospun nanofibrous membrane as an efficient probe for visual colorimetric detection of TNT^33^ and Hg^2 + ^34. We speculated that the incorporation of active probes inside NF matrix might be playing a role in preventing the diffusion of analytes into the NF and thereby, access the analyte, further decreasing the sensitivity of the overall sensor performance. In order to rule out this possibility, we introduced the integration of binding sites in a manner that allows more direct interaction between the probe and analyte by depositing AuNC onto the NF surface. The anchored AuNC on the electrospun NF can effectively adsorb analytes directly onto their surfaces, leading to superior sensing ability. Thus, the most favorable morphologies have been identified and enhanced Hg^2+^ sensing performance has been rendered in the present investigation. The construction of composites from fluorescent AuNC and electrospun NF allows one to achieve great versatility in combining and enhancing their valuable properties with a tremendous increase in their detection limit allowing parts per trillion (ppt) level detection. To the best of our knowledge, there has not been much report to study on the real time monitoring of Hg^2+^ detection by using fluorescent electrospun NF incorporating AuNC. Additionally, real time monitoring approach affirms the rapid readability of the responses.

## Results

### Controlled assembly of gold nanoclusters (AuNC) on the PCL nanofiber (PCL-NF) surface

**Figure 1:**
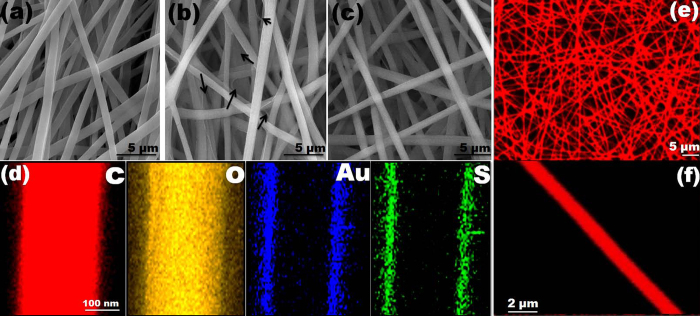
Representative SEM images of the electrospun PCL-NF (**a**) gold nanocluster (AuNC) coated PCL-NF termed as AuNC*PCL-NF, in the presence (**b**) and absence (**c**) of excess BSA ligand. The arrow indicates the excess amount of weakly adsorbed ligand across the NF surface. (**d**) HAADF-STEM elemental mapping of the C, O, S and Au elements present in the AuNC*PCL single NF, shows AuNC are consistently anchored on the surface of NF. (**e**) Fluorescence image of AuNC*PCL-NF taken using a CLSM, excited at 488 nm. The emitted red color owing to the characteristics emission of AuNC (**f**) CLSM image of the AuNC*PCL single NF, confirms the uniform bright fluorescent feature throughout the NF surface.

The method here involves the anchoring of fluorescent BSA capped AuNC on the electrospun PCL-NF surface, named as AuNC*PCL-NF for real time monitoring of Hg^2+^ detection. The schematic representation of experimental procedures is given in Fig. S1. The fluorescent AuNC is prepared based on reported procedure^35^ and subsequently decorated on the surface of the electrospun PCL-NF by dip coating method. The morphological description of a fashioned AuNC*PCL-NF is illustrated in Fig. 1. By controlling the electrospinning process parameter, randomly oriented bead-free uniform PCL-NF with an average diameter of 280 ± 40 nm has been obtained as depicted in Fig. 1a. The morphology of the as-spun PCL-NF has been found to be smooth whereas, it became much rougher after anchoring the AuNC on their surface and the diameter has been slightly increased by few tens of nanometers compared with that of the pristine NF (Fig. 1b-c and S2).

The mechanism for anchoring fluorescent AuNC on the surface of PCL-NF might be attributed to the formation of hydrogen bonding between the carbonyl group in the PCL-NF and the carboxylic acid groups capped on the surface of the AuNC, as presented in Fig. S1c. Moreover, the individual AuNC are also attracted each other via interaction between the COOH groups present on the surface of AuNC which facilitates a way to form a close proximity of AuNC on the surface of the NF^36^. The proximity rate depends on the coating time which clearly given in Fig. S3. In addition, slight swelling nature of the PCL-NF when it got immersed into the AuNC solution facilitates more adsorption of AuNC on their surface. Further, it has been observed that, with the complete evaporation of the solvent, the adsorbed AuNC have been found to be closely packed and more compacted, as the NF shrinks.

Effective control of the coating density of AuNC on the NF surface can be carried forward by controlling the processing time involved and their correlated enhanced emission characteristics are presented in Figs. S3 and S4. When the incubation time increased from 12 to 24 hours (Fig. S3 g–h), more AuNC have been found to be adsorbed onto the PCL-NF forming a patch-like structure. As seen in emission spectra, the intensity has been observed to reach a maximum peak at 2 hours dipping and no more increasing beyond that has been found, suggesting the saturation time for obtaining the optimal AuNC*NF surface to be ≥2 hours. Upon increasing the coating time further, a more broadened emission peak has been speculated further leading to a red shift in the spectra, due to increasing intercoupling as well aggregation of the AuNC. In addition, as seen from the 24 hours processed sample of confocal laser scanning microscopy (CLSM) image, a few brighter red spots indicating some number of aggregated AuNC on the NF surface has been noticed (Fig. S5). The thin layer of ligand non-specifically adsorbed on the optimized NF surface besides the BSA binding with AuNC, forming bridges between the NF has been clearly illustrated in Fig. 1b. It has been noted that the presence of excess ligand has a decreasing effect on the binding ability with analytes and thereby the diffusion potential into the interior surface of the NF. In addition, we also have speculated the capability of the excess ligand to actively react with other metal ions^37^,^38^. Therefore, a ligand extraction procedure has been adopted to remove the substantial fraction of the weakly adsorbed excess ligand from the supported AuNC, since the ligand is readily soluble in water.

The compared fluorescence features of AuNC*PCL-NF is illustrated in Fig. S6. Immediate detachment of AuNC from the NF surface has been found when the coating time is low (10 minutes to 2 hours) suggesting likely incidences of adsorption instead of attachment, with the fluorescence intensity reaching maximum. The outcome of the result suggested that 3 hours of coating time following 30 minutes of washing time has been good enough to remove the excess amount of ligand. The result suggested that the significant removal of ligand has a narrower emission profile relative to unremoved one. Concluding from the observed results, the ligand removal procedure removes a substantial fraction of the stabilizing BSA molecules from the supported AuNC, but does not affect their morphology and fluorescence properties. Specifically, the densely packed AuNC on the NF surface might have visible voids because of the ligand effect which facilitate rapid diffusion of analytes into and out of the surface. Additionally, it has been discerned that the AuNC are not detached from the surface of NF after dipping in water for a prolonged period of time, say 12 to 24 hours.

The additional advantage here has been nil surface functionalization required for sensing Hg^2+^, simplifying the process on a larger scale. As it can be seen from the high-angle annular dark- field scanning transmission-electron microscopy (HAADF-STEM) elemental mapping, the AuNC have consistently been anchored on the surface of PCL-NF (Fig. 1d). The observed results provide the clear evidence that AuNC are exclusively coated outer layer of the NF surface and not embedded inside the fiber matrix.

The emission properties of the NF have been further explored by CLSM. The CLSM image of the AuNC*PCL-NF shows a characteristic red emission owing to the AuNC anchored on its surface as shown in Fig. 1e. The characteristic red emission of the AuNC have been preserved after decorating on the NF surface and the resulting brighter images from the enhanced scattering characteristics of NF with higher AuNC surface coverage^39^,^40^,^41^. Moreover, the enhanced brightness can be attributed to the closer inter particle spacing between constituent AuNC and reduced ligand effect on the emission^42^. Further, the broad emission features of the AuNC*PCL-NF have also been confirmed by observing the images at different emission wavelengths by tuning the detector 505–794 nm (Fig. S7).

In the further course of our experiments, we investigated the stability over an extended time period at ambient condition to explore the resultant AuNC*PCL-NF for real time monitoring of Hg^2+^ detection. A worthwhile mention has to be briefed about their stability for several months without self quenching, eliminating the limitations arising from aggregation-induced self-quenching and photo bleaching (see Fig. S8). In addition, the stability of AuNC*PCL-NF have been tested towards various pH values. The observed results confirmed that there were no considerable changes in their fluorescence intensity as shown in Fig. S9. Therefore, the performance and efficiency of AuNC*PCL-NF based sensor doesn’t vary at various pH levels. The well defined characteristics of the single nanofiber (SNF) enable their facile identification and make them a reliable medium to study the single NF-based analyte over single particle-based sensor located by CLSM (Fig. 1f).

### Sensor performance

**Figure 2:**
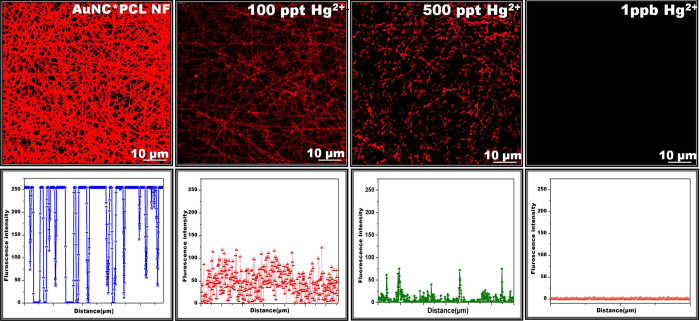
Sensing performance of AuNC*PCL-NF towards Hg^2+^. CLSM images of AuNC*PCL-NF before and after exposure to various concentration of Hg^2+^ and their line scanning profiles, recorded across the each spot. The gradual decreases of fluorescence in the AuNC*PCL-NF upon increasing the concentration of Hg^2+^ were noticed. The formation of fiber junctions during the electrospinning process limits the diffusion of mercury ions between these junctions, thereby few number of brighter spots are visually seen in 500 ppt Hg^2+^ treated AuNC*PCL-NF.

The sensing performance of AuNC*PCL-NF towards Hg^2+^ have been evaluated by immersing the membrane in Hg^2+^ containing water for 10 minutes and then their corresponding fluorescence spectra have been recorded as shown Fig. S10. The gradual decreases in fluorescence intensity upon increasing concentrations of Hg^2+^ have been observed and at higher concentration (~ppm), the spectra have been blue shifted which might be due to the strong interaction between gold and mercury. The lower detection limit has been found to be 50 ppt. Further, the sensing performance of AuNC*PCL-NF have been tested upon exposure to various concentrations of 2 μL of aqueous Hg^2+^ on the NF surface and their corresponding fluorescence image, spectra have been recorded using CLSM. Before adding the Hg^2+^ solution, the CLSM image of AuNC*PCL-NF exhibited bright and uniform fluorescence features and their observed intensity has been taken as a control. As evident from the CLSM image, the fluorescence intensity gradually decreased upon increasing Hg^2+^ ion concentration (100 ppt to 1 ppb) and their collected intensity data across the NF surface has confirmed their uniform response, visually illustrated in Fig. 2. The uniform sensing response of AuNC*PCL-NF ensembles evidenced that each SNF can potentially act as an independent sensor, providing a possibility to miniaturize the materials involved and the overall cost. Following this, the experiment has been taken further to the SNF level. The detection limit of the SNF-based sensor has been observed to go down to ppt level.

**Figure 3:**
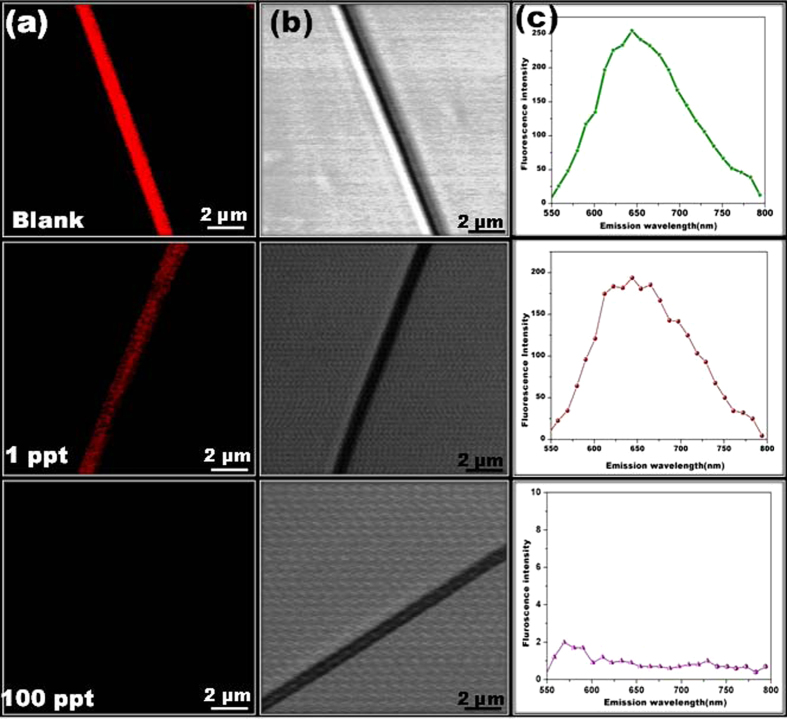
(**a**) CLSM images of AuNC*PCL-SNF before and after addition of Hg^2+^ (100 ppt and 1 ppt) solution and their corresponding DIC image is presented in Figure. (**b**) The result shows that there is no change in their morphology and only the fluorescence feature of the NF changed according to the concentration. (**c**) The resultant fluorescence spectra collected from the surface of the NF.

Upon exposure of 100 ppt Hg^2+^, the fluorescence intensity of the AuNC*PCL-SNF completely disappeared, whereas upon introduction of 1ppt, considerable response has been noticed following the collection of the spectra, as illustrated in Fig. 3. The differential interference contrast (DIC) image confirms no obvious changes in the NF morphology identified in the presence of Hg^2+^. The important notable thing here is that there was no considerable change in emission features when the concentration of Hg^2+^ was below 100 ppt in bulk ensembles of NF, thus indicating the limit of Hg^2+^ detection. The observed difference in the sensing performance of NF ensemble might be due to the limited diffusion ability of mercury ions into interior surface of the membrane. Uniform decreases in the emission intensity along the NF mark a significant development of a reliable method for real time application.

To demonstrate the practical application of developed probe i.e. AuNC*PCL-NF, we have tested the Hg^2+^ in tap water. The collected tap water is further filtered through a 0.2 μm membrane and then analyzed by using ICP-MS. The observed results confirm that the collected tap water didn’t contain Hg^2+^. Therefore, various concentrations of Hg^2+^ in tap water were prepared by using stock solution. There was no considerable change in the fluorescence spectra of AuNC*PCL-NF when treated with tap water, confirmed the usability of our designed probe in practical applications. The detection limit has also been to be as ~50 ppt indicating that the efficiency of the probe is independent from the matrix effect of the tap water as illustrated in Fig. S11.

### Specific selectivity towards Mercury

**Figure 4:**
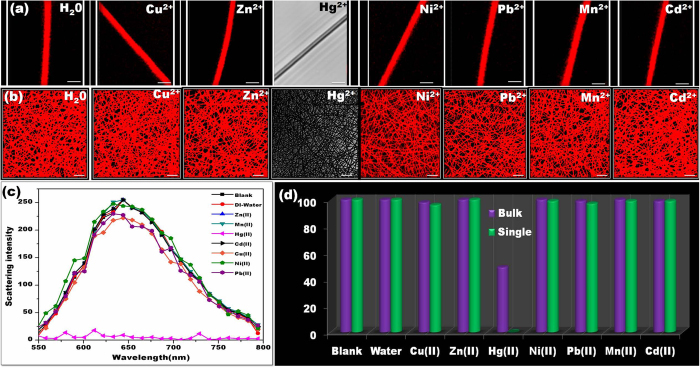
Selective sensing performance of AuNC*PCL-SNF and AuNC*PCL-NF mat. CLSM image presents the fluorescence response of AuNC*PCL-SNF (**a**) and AuNC*PCL-NF mat (**b**) to various metal ions (indicated in each image) at a concentration of 10 ppm. (Scale bar Figure a-2 μm and b-5 μm) (Note: For Hg^2+^ only, DIC images are given since fluorescence is completely quenched and CLSM image become full black). The H_2_O treated AuNC*PCL-NF shows their stability as well proves the observed decreased fluorescence upon addition of metal ions is not because of solvent. (**c**) Variation in the emission spectra of different metal ions treated AuNC*PCL-SNF (**d**) Bar diagram illustrating the relative variation in the fluorescence intensity of the single NF and nanofibrous mat.

Further, we have investigated the selectivity and specificity of the sensor in the presence of various competing metal ions such as Cu^2+^, Ni^2+^, Mn^2+^, Zn^2+^, Cd^2+^, and Pb^2+^ at a concentration of 10 ppm and volume of 5 μL, for both bulk ensemble and SNF. As shown in Fig. 4a-b, there has been apparent disappearance of fluorescence intensity upon addition of Hg^2+^, whereas no obvious changes in the presence of water and other metal ions have been noticed. The recorded fluorescence scattering spectra of AuNC*PCL-SNF shows a huge decrease in the intensity merely for Hg^2+^ compared to other metal ions (Fig. 4c). The variation in the relative fluorescence intensity of the SNF and bulk NF ensemble is presented in Fig. 4d. Further, the effect of salts (sodium, potassium and calcium) and biothiols such as cysteine (Cys), glutathione (GSH) were also investigated. As expected, there were no accountable change observed in the fluorescence intensity of AuNC*PCL-NF visually as well as spectroscopically as illustrated in Fig. S12 and Fig. S13, respectively. The attained specific selectivity towards Hg^2+^ over competent Cu^2+^ ions has been achieved effectively in the absence of excess amount of ligand on the NF surface. To confirm this scenario, we have studied and compared the enhancement of selectivity towards Cu^2+^ by unwashed AuNC*PCL-NF, upon introduction of 10 ppm Cu^2+^ and their outcome (Fig. S14) suggested that there is a decrease in the fluorescence intensity. The observed results clearly demonstrate that the ligand removal step effectively enhances the selectivity without greatly altering their parent features.

### Real time monitoring

**Figure 5:**
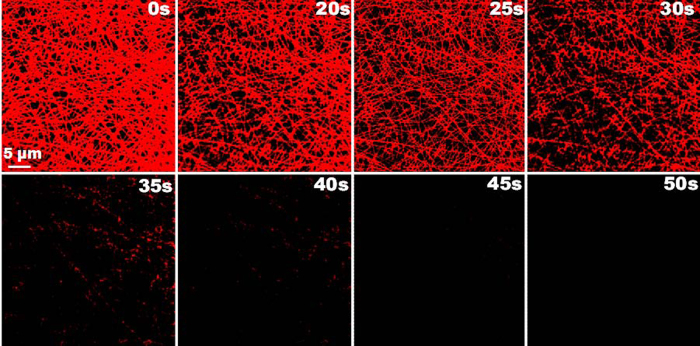
Real time visual monitoring of Hg^2+^ detection. Time dependent CLSM images of AuNC*PCL-NF upon addition of 1 ppb Hg^2+^. The attained AuNC*PCL-NF sensor system here reached the basic requirement since maximum permissible limit of mercury in drinking water is 2 ppb set by US Environmental Protection Agency (EPA). The changes in their fluorescence intensity are clearly visible and all the images have same scale bar (5 μm).

**Figure 6:**
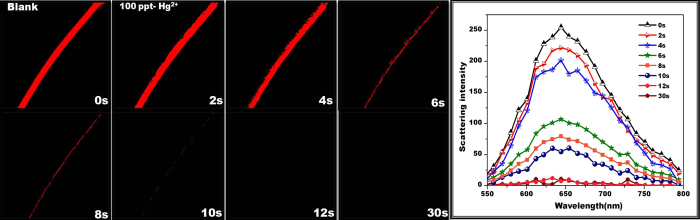
Real time visual monitoring of Hg^2+^ detection. Time dependent CLSM images and their corresponding spectra of AuNC*PCL-SNF upon addition of 100 ppt Hg^2+^. [Note: An immediate disappearance of fluorescence has been observed when the Hg^2+^ solution is placed directly on a SNF. To overcome this, we have performed real time monitoring by placing the Hg^2+^ solution near the surface of the SNF and frequently capture the image every second. The solution slowly contacted near the surface of NF, following which decreased fluorescence intensity has been observed resulting in complete disappearance after 10 seconds].

Going a step ahead, we have also performed the real time detection of Hg^2+^ since response time is a key factor in evaluating the practical application of a sensor. Firstly, making it certain that the observed response has been due to specific binding with mercury and not with water, we have evaluated supplementary control experiments with water. As expected, no change in the fluorescence intensity has been observed when treated with water (3 μL, see Fig. S15) and even higher volume (6 μL, see Fig. S16). As shown in Fig. 5, after 2 μL of 1 ppb Hg^2+^ introduced (at t =1s), a gradual decrease of fluorescence intensity has been noticed corresponding to an increasing time. The majority of intensity decreased within 30 seconds suggesting a faster response time with low amount of analyte solution. More interestingly, an immediate disappearance of fluorescence has been observed when the Hg^2+^ solution is directly placed on a SNF. To overcome this, we have performed real time monitoring by placing the Hg^2+^ solution near the surface of the SNF and frequently capture the image every second. The solution slowly contacted near the surface of SNF, following which decreased fluorescence intensity has been observed resulting in complete disappearance after 10 seconds. The conspicuous changes in the spectrum profile as well their images have been recorded and presented in Fig. 6. As compared with bulk NF ensembles, the SNF have reported faster response time. In order to visualize the effect of Hg^2+^ on the morphology of AuNC*PCL-NF in real time, 1 ppm Hg^2+^ had been exposed on their surface with the DIC images captured simultaneously (see Fig. S17).

The obtained results suggest no degradation in the integrity of the NF during the sensing experiment. In addition, no detectable aggregation of Au-Hg has been seen. Therefore, it might be expected to observe uniform adsorption on their surface, which will be investigated in the future section. We were further intended to study the real time detection of sensor with different metal ions. Taking forward, the study was carried out for Cu^2+^ wherein unlike Hg^2+^, the excess amount of Cu^2+^ (5 μL, 10 ppm) had been exposed and their corresponding detection has been monitored every 5 seconds as illustrated in Fig. S18. The characteristic emission devoid of any quenching has found to be retained for 35 seconds. The real time detection have also been performed with 10 ppm Pb^2+^ (Fig. S19) and 10 ppm Zn^2+^ (Fig. S20). The observed result again proves the selectivity even at higher concentration and reaction time. These results clearly demonstrate that the optimized system has been well suited for real-time monitoring of selective Hg^2+^ detection.

### Mechanism of enhanced sensitivity and selectivity

Despite the numerous studies on the AuNC based mercury sensor, the sensing mechanism still goes far from being understood. Previous reports by several groups have shown that the sensing performance of AuNC has a direct relation to the aggregation induced fluorescence quenched mechanism^43^,^44^,^45^,^46^,^47^,^48^,^49^,^50^,^51^. Recent studies, however, suggest a contrary mechanism for the same, wherein the quenching has been accomplished by the complex formation between BSA–gold nanoclusters^52^,^53^. Unlike the reported mechanism so far, the fundamental sensitivities of our Hg^2+^ sensors might not directly related to the aggregation induced fluorescence quenching, since they are stably immobilized on the surface of the NF and also binding ability of BSA is almost same for nearly competent metals ion present in water.

**Figure 7:**
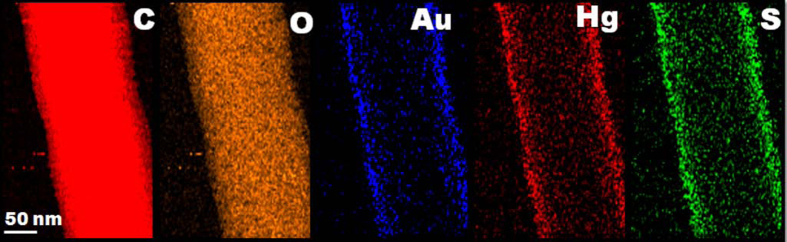
EDX mapping of C, O, S, Au and Hg elements presents in the AuNC*PCL-SNF confirms that the mercury is adsorbed on the NF surface.

We further speculated that the selective sensing mechanism might be due to the enhanced interaction between gold and mercury, resulting in rapid adsorption followed by the formation Au-Hg amalgam^54^,^55^,^56^.The performed EDX elemental mapping of 1 ppm Hg^2+^ (~1 minute) solution exposed AuNC*PCL-SNF revealed the uniform adsorption of mercury ions on the entire length of the NF surface (Fig. 7). The adsorbed Hg^2+^ ions has been reduced to form Hg^0^ by the AuNC*PCL-NF and the observed XPS spectrum of 4f^7/2^ at 100.87 eV has also been confirmed as illustrated in Fig. S21. Unlike previous studies, the adsorbed mercury ions does not trigger the release from the surface, but naturally prompt more adsorption and form a complex, confirmed by EDX mapping. Our suspicion around the formation of Au-Hg amalgam rather than mere adsorption around the surface of the AuNC led to monitor the growth of Au-Hg at extended reaction time period of 3 minutes.

**Figure 8:**
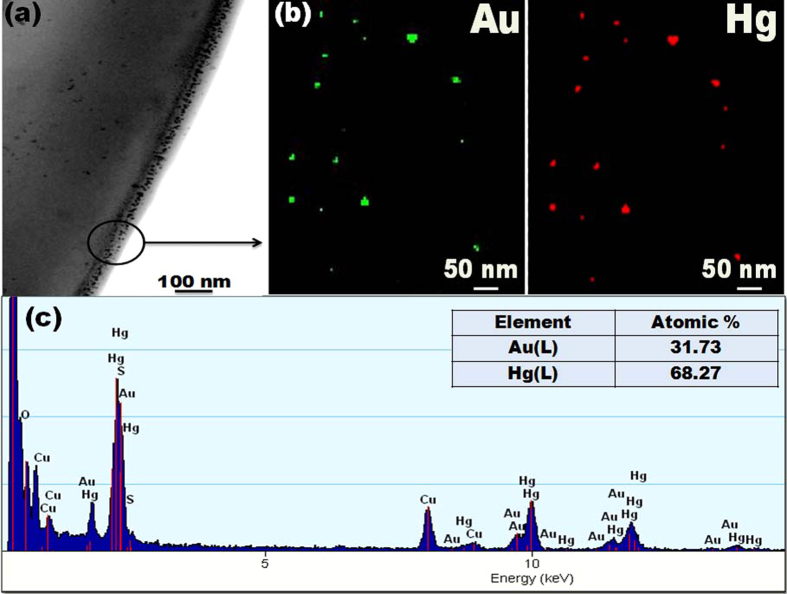
Characterization of formation of the Au-Hg amalgam. (**a**) STEM-HAADF image of the 1 ppm Hg^2+^ treated (~2 minutes) AuNC*PCL-SNF. (**b**) EDX elemental mapping analysis of single Au-Hg particle for Au and Hg confirms that the formed particles are Au-Hg amalgam. Mercury is only located on the Au at the sites of the AuNC*PCL-SNF, confirming the interaction between gold and mercury (**c**) EDX spectrum of Au-Hg amalgam.

The STEM-HAADF image (Fig. 8a) and elemental mapping of the formed Au-Hg particles revealed the uniform distribution of Au and Hg ions, confirming the structure of Au-Hg amalgam. As seen from Fig. 8b, mercury has been located on the NF surface at the site of AuNC, confirming strong Au and Hg interaction. Moreover, EDXS analysis (see Fig. 8c) confirmed the presence of two elements in the formed nanoparticles (31.73 atom% Au and 68.27 atom% Au). Careful observation clearly pointed out the non-uniform diameters of the Au-Hg composites and their larger size than that of the AuNC.

The combined observations of XPS and EDX mapping proved the formation of Au-Hg amalgam followed by the rapid adsorption of mercury ions along with the ligand. The attained enhancement in the sensing performance is not exclusively dependent on the AuNC. It has been well known that the amino groups have tendency to bind with the metal ions. Therefore, it would be expected here that the amino acids present in the BSA provoke the rapid adsorption of Hg^2+^ ions and continuously trigger them to bind tightly to each other. Owing to the formation of Au-Hg amalgam, the reusability of the fabricated sensor device is limited.

## Conclusion

Herein, a successful demonstration of fluorescent AuNC coated on the surface of the electrospun polymeric NF has been shown, along with the application of the sensing probe to monitor the mercury detection in real time. Interestingly, the AuNC*PCL-NF nanofibrous structure has made this effort possible for handling the sensor probes in a more straightforward manner. Further, this has also enhanced the accessibility of the active site resulting in a fast and efficient response time. An significant improvement in this research has been the usage of a single NF for the detection of Hg^2+^, acknowledging the miniaturization of sensor devices. The prepared AuNC*PCL-NF, to be free of excess ligand, prioritized the selective Hg^2+^ over competent metal ions such as Cu^2+^, Ni^2+^, Mn^2+^, Zn^2+^, Cd^2+^, and Pb^2+^ present in the water. The detailed mechanism involving the sensing performance has been well studied. The EDXS, elemental mapping and XPS analyses have proven the formation of Au-Hg amalgam followed by the rapid adsorption of mercury ions along with the ligand. Inspiringly, considering all aspects detailed, we strongly observe and believe that this paper represents advancement in the field of sensors for three reasons (i) real time monitoring of Hg^2+^ detection (ii) enhanced selectivity towards Hg^2+^ over competent Cu^2+^ ions in water (iii) explored detailed mechanistic investigation. These observations hope to prove helpful and serve as the bottomline for new fluorescent single NF based sensors, expected to bring drastic advances in water quality monitoring and extended in principle to other metal ions.

## Experimental details

### Materials

Tetrachloroauric acid trihydrate (HAuCl_4_.3H_2_O), bovine serum albumin (BSA), polycaprolactone (PCL) (Mw: 80,000, Sigma Aldrich), L-Cysteine, Glutathione, mercuric (II) acetate, zinc (II) acetate, manganese (II) acetate, lead (IV) acetate, cadmium (II) acetate and copper acetate (II) were purchased from Sigma–Aldrich. Sodium hydroxide pellet was obtained from Merck. All chemicals used were of analytical grade and were used without further purification. Deionized water, N,N-dimethylformamide (DMF) (Riedel, Pestanal), dicholoromethane (DCM) (Sigma, Extra Pure) were used as solvent. AVS TITRINORM (pH~4), Phosphate buffered saline (pH~7) and Tris buffer (pH~9) were used as a buffer solution.

### Preparation of fluorescent gold nanoclusters (AuNC)

The fluorescent gold nanocluster was prepared according to the previously reported method^35^. In brief, 10 mM of HAuCl_4_ solution (10 mL) was mixed with equal amounts of BSA solution (50 mg mL^-1^) at 37 °C under vigorous stirring. Two minutes later, 1 mL of 1 M, NaOH solution was introduced into the mixture, and the reaction was allowed to proceed under vigorous stirring at 37 °C for 12 h. The prepared gold nanocluster emitted red color when it exposed under UV lamp, which will be further decorated on the NF surface.

### Preparation of electrospun PCL nanofibers (NF)

The clear and homogenous PCL solution was prepared in DMF/DCM (3:1, v/v) solvent system, using 18% (w/v, with respect to solvent) polymer concentration. The PCL solutions were taken up in a 5 mL syringe fitted with a metallic needle of 0.4 mm inner diameter. The syringe was fixed horizontally on the syringe pump (model KDS-101, KdScientific, USA). The electrode of the high-voltage power supply (Spellman, SL30, USA) was clamped to the metal needle tip, and the plate-shaped aluminum collector was grounded. Electrospinning parameters were adjusted as follows: feed rate of solutions =0.5 mL/h, applied voltage =10 kV, tip-to-collector distance =10 cm. The electrospinning process was performed at 25 °C in a Plexiglas box. After the electrospun NF were deposited on the glass substrate, they were kept in a vacuum oven overnight to remove the solvent residual if present in the NF. In order to isolate single NF, the spinning process was performed for few seconds.

### Preparation of fluorescent BSA capped AuNC decorated PCL-NF (AuNC*PCL-NF)

The electrospun PCL-NF was immersed in a BSA capped gold nanocluster solution at different time period. Followed this, the membrane was separated from the solution carefully and dried in air at room temperature. Based on various characterization tools (SEM, Fluorescence spectra and CLSM images), the optimal coating time has been fixed for three hours. Further, the thin layer of ligand non-specially adsorbed on the surface of the NF which might expected to reduce the quenching efficiency. Therefore, ligand extraction procedure has been adopted to remove the weakly adsorbed excess ligand from the surface of NF. For this, the membrane was washed with water with shaking for 30 minutes and then dried at room temperature. The prepared sample was named as AuNC*PCL-NF. The same procedure has been followed for single NF.

### Characterizations

The morphology and diameter of the NF at various stages were measured by scanning electron microscope (SEM, Quanta 200 FEG) and transmission electron microscopy (TEM, Tecnai G2 F30). The chemical composition of the AuNC*PCL-NF were performed using high-performance X-ray photoelectron spectroscopy (XPS, Thermo K-alpha-monochromated). Fluorescence emission spectra were measured by time-resolved fluorescence spectrophotometer (FL-1057 TCSPC). Confocal laser scanning microscopy (CLSM, Zeiss LSM 510) images were recorded where excitation sources were fixed at 488 nm for all experiments and the images were captured at 20x magnification. The presence of Hg^2+^ in tap water is studied using Inductively Coupled Plasma-Mass Spectrometry (ICP-MS, Thermo, X Series II).

### Confocal laser scanning microscopy imaging

The PCL-NF were deposited on microscopic glass slides and the AuNC were coated carefully on their surface. Following this, the excess amount of ligands was carefully removed by gentle washing with water. The excitation source was fixed at 488 nm and the images were captured at 20x magnification. The pristine PCL-NF were considered as negative control:  in brief, to adjust all the parameters including the laser intensity and gain, until fluorescent signals were out of view from the pure PCL-NF sample; following which the same parameters were used to observe the AuNC coated NF.

### Detection of metal ions in water

A stock solution of 100 ppm containing different metal ions (Hg^2+^, Cu^2+^, Ni^2+^, Mn^2+^, Zn^2+^, Cd^2+^, and Pb^2+^) were prepared in de-ionized water, further diluted to different concentrations according to the need of the experiment. The sensing performance was carried by dropping the 2 μL of the desired concentration of the metal ion solution on the NF surface. The CLSM images were taken after drying the NF. For real time application, the images were taken immediately after the passing of the metal ion on the NF and their corresponding spectra also recorded as a function of time.

## Additional Information

**How to cite this article**: Senthamizhan, A. *et al*. Real-time selective visual monitoring of Hg^2+^ detection at ppt level: An approach to lighting electrospun nanofibers using gold nanoclusters. *Sci. Rep.* **5**, 10403; doi: 10.1038/srep10403 (2015).

## Supplementary Material

Supplementary Information

## References

[b1] Zahir F., Rizwi S. J., Haq S. K. & Khan R. H. Low Dose Mercury Toxicity and Human Health. Environ. Tox. Pharm. 20, 351–360 (2005). 10.1016/j.etap.2005.03.007 21783611

[b2] Orozco J. *et al.* Artificial Enzyme-Powered Microfish for Water-Quality Testing. ACS Nano 7, 818–824 (2013). 23234238 10.1021/nn305372n

[b3] Hoang C. V., Oyama M., Saito O., Aono M. & Nagao T. Monitoring the Presence of Ionic Mercury in Environmental Water by Plasmon-Enhanced Infrared Spectroscopy. Sci. Rep. 3, (2013) (10.1038/srep01175). PMC3569543 23405272

[b4] Wang L., Yao T., Shi S., Cao Y. & Sun W. A label-free fluorescent probe for Hg^2+^ and biothiols based on graphene oxide and Ru-complex. Sci. Rep. 4, (2014) (10.1038/srep05320). PMC4060466 24936798

[b5] An J. H., Park S. J., Kwon O. S., Bae J. & Jang J. High-Performance Flexible Graphene Aptasensor for Mercury Detection in Mussels. ACS Nano 7, 10563–10571 (2013). 24279823 10.1021/nn402702w

[b6] Wei Q. *et al.* Detection and Spatial Mapping of Mercury Contamination in Water Samples Using a Smart-Phone. ACS Nano 8, 1121–1129 (2014). 24437470 10.1021/nn406571t PMC3949663

[b7] Ke J., Li X., Zhao Q., Hou Y. & Chen J. Ultrasensitive Quantum Dot Fluorescence quenching Assay for Selective Detection of Mercury Ions in Drinking Water, Sci. Rep. 4, (2014) (10.1038/srep05624). PMC4087922 25005836

[b8] Guo Z. *et al.* A molecular-gap device for specific determination of mercury ions. Sci. Rep. 3, (2013) (10.1038/srep03115). PMC3814579 24178058

[b9] Kim H. N., Ren W. X., Kim J. S. & Yoon J. Fluorescent and colorimetric sensors for detection of lead, cadmium, and mercury ions. Chem. Soc. Rev. 41, 3210–3244 (2012). 22184584 10.1039/c1cs15245a

[b10] Xie J., Zheng Y. & Ying J. Y. Highly Selective and Ultrasensitive Detection of Hg^2+^ Based on Fluorescence Quenching of Au Nanoclusters by Hg^2+^-Au^+^ Interactions. Chem. Comm. 46, 961–963 (2010). 20107664 10.1039/b920748a

[b11] Song Y. *et al.* Identification of single nanoparticles. Nanoscale 3, 31–44 (2011). 20949213 10.1039/c0nr00412j

[b12] Li H. & Rothberg L. Colorimetric detection of DNA sequences based on electrostatic interactions with unmodified gold nanoparticles. PNAS 101, 14036–14039 (2004). 15381774 10.1073/pnas.0406115101 PMC521116

[b13] Bera K., Das A. K., Nag M. & Basak S. Development of a Rhodamine−Rhodanine-Based Fluorescent Mercury Sensor and Its Use to Monitor Real-Time Uptake and Distribution of Inorganic Mercury in Live Zebrafish Larvae. Anal. Chem. 86, 2740–2746 (2014). 24559034 10.1021/ac404160v

[b14] Tan H., Liu B. & Chen Y. Lanthanide Coordination Polymer Nanoparticles for Sensing of Mercury(II) by Photoinduced Electron Transfer. ACS Nano 6, 10505–10511 (2012). 23121519 10.1021/nn304469j

[b15] Long F., Zhu A., Shi H., Wang H. & Liu J. Rapid on-site/*in situ* detection of heavy metal ions in environmental water using a structure-switching DNA optical biosensor. Sci. Rep. 3, (2013) (10.1038/srep02308). PMC3725506 23892693

[b16] Wan W., Biyikal M., Wagner R., Sellergren B. & Rurack K. Fluorescent Sensory Microparticles that “Light-up” Consisting of a Silica Core and a Molecularly Imprinted Polymer (MIP) Shell. Angew. Chem. Int. Ed. 52, 7023–7027 (2013). 10.1002/anie.201300322 23716378

[b17] Tam J. M. *et al.* Controlled Assembly of Biodegradable Plasmonic Nanoclusters for Near-Infrared Imaging and Therapeutic Applications. ACS Nano 4, 2178–2184 (2010). 20373747 10.1021/nn9015746 PMC2862619

[b18] Uzun A., Ortalan V., Hao Y., Browning N. D. & Gates B. C. Nanoclusters of gold on a high-area support: almost uniform nanoclusters imaged by scanning transmission electron microscopy. ACS Nano 3, 3691–3695 (2009). 19863069 10.1021/nn9008142

[b19] Zhangm H. & Cui H. High-density assembly of chemiluminescence functionalized gold nanodots on multiwalled carbon nanotubes and their application as biosensing platforms. Nanoscale 6, 2563–2566 (2014). 24457618 10.1039/c3nr05574d

[b20] Song H. D. *et al.* On-Chip Colorimetric Detection of Cu^2+^ Ions via Density-Controlled Plasmonic Core−Satellites Nanoassembly. Anal. Chem. 85, 7980–7986 (2013). 23865681 10.1021/ac401796q

[b21] Lin Z. *et al.* Recyclable fluorescent gold nanocluster membrane for visual sensing of copper(II) ion in aqueous solution. Analyst. 137, 2394–2399 (2012). 22489283 10.1039/c2an35068h

[b22] Su L. *et al.* Immobilization of bovine serum albumin-protected gold nanoclusters by using polyelectrolytes of opposite charges for the development of the reusable fluorescent Cu^2+^-sensor. Biosens. Bioelectron. 44, 16–20 (2013). 23384766 10.1016/j.bios.2013.01.005

[b23] Chen L. Y., Ou C. M., Chen W. Y., Huang C. C. & Chang H. T. Synthesis of Photoluminescent Au ND−PNIPAM Hybrid Microgel for the Detection of Hg^2^^+^. ACS Appl. Mater. Interfaces 5, 4383–4388 (2013). 23618348 10.1021/am400628p

[b24] Choi Y., Park Y., Kang T. & Lee L. P. Selective and sensitive detection of metal ions by plasmonic resonance energy transfer- based nanospectroscopy. Nat. Nanotechnol. 4, 742–746 (2009). 19893511 10.1038/nnano.2009.258

[b25] Wang X. *et al.* Electrospun Nanofibrous Membranes for Highly Sensitive Optical Sensors. Nano Lett. 2, 1273–1275 (2002).

[b26] Cai Y., Yan L., Liu G., Yuan H. & Xiao D. *In situ* synthesis of fluorescent gold nanoclusters with electrospun fibrous membrane and application on Hg (II) sensing. Biosens. Bioelectron. 41, 875–879 (2013). 23021839 10.1016/j.bios.2012.08.064

[b27] Si Y. *et al.* Optimized colorimetric sensor strip for mercury(II) assay using hierarchical nanostructured conjugated polymers. J. Mater. Chem. A. 2, 645–652 (2014).

[b28] Wang W. *et al.* Colorimetric and fluorescent nanofibrous film as a chemosensor for Hg^2+^ in aqueous solution prepared by electrospinning and host-guest interaction. Chem. Comm. 48, 6040–6042 (2012). 10.1039/c2cc17664e 22334338

[b29] Li Y. *et al.* Colorimetric sensor strips for lead (II) assay utilizing nanogold probes immobilized polyamide-6/nitrocellulose nano-fibers/nets. Biosens. Bioelectron. 48, 244–250 (2013). 23707870 10.1016/j.bios.2013.03.085

[b30] Orriach-Fernández F. J. *et al.* A sensing microfibre mat produced by electrospinning for the turn-on luminescence determination of Hg^2+^ in water samples. Sensor Actuat B-Chem. 195, 8–14 (2014).

[b31] Ongun M. Z. *et al.* Determination of Hg(II) at sub-nanomolar levels: A comparative study with nanofibrous materials and continuous thin films. Sensor Actuat B-Chem.181, 244–250 (2013).

[b32] Amna T. *et al.* Camptothecin loaded poly(-caprolactone)nanofibers via one-step electrospinning and their cytotoxicity impact. *Colloids and Surfaces A: Physicochem*. Eng. Aspects 431, 1–8 (2013).

[b33] Senthamizhan A., Celebioglu A. & Uyar T. Ultrafast on-site selective visual detection of TNT at Sub ppt level using fluorescent gold cluster incorporated single NF. Chem. Comm. 51, 5590–5593 (2015). 24949681 10.1039/c4cc01190b

[b34] Senthamizhan A., Celebioglu A. & Uyar T. Flexible and highly stable electrospun nanofibrous membrane incorporating gold nanocluster as a efficient probe for visual colorimetric detection of Hg(II). J. Mater. Chem. A. 2, 12717–12723 (2014).

[b35] Xie J., Zheng Y. & Ying J. Y. Protein-Directed Synthesis of Highly Fluorescent Gold Nanoclusters. J. Am. Chem. Soc. 131, 888–889 (2009). 19123810 10.1021/ja806804u

[b36] Dong H., Wang D., Sun G. & Hinestroza J. P. Assembly of Metal Nanoparticles on Electrospun Nylon 6 Nanofibers by Control of Interfacial Hydrogen-Bonding Interactions. Chem. Mater. 20, 6627–6632 (2008).

[b37] Li X., Zhang S., Kulinich S. A., Liu Y. & Zeng H. Engineering surface states of carbon dots to achieve controllable luminescence for solid-luminescent composites and sensitive Be^2+^ detection. Sci. Rep. 4, (2014) (10.1038/srep04976).

[b38] Durgadas C. V., Sharma C. P. & Sreenivasan K. Fluorescent gold clusters as nanosensors for copper ions in live cells. Analyst 136, 933–940 (2011). 21152627 10.1039/c0an00424c

[b39] Hwang E., Smolyaninov I. I. & Davis C. C. Surface Plasmon Polariton Enhanced Fluorescence from Quantum Dots on Nanostructured Metal Surfaces. Nano Lett. 10, 813–820 (2010). 20112921 10.1021/nl9031692

[b40] Sun Y. & Xia Y. Increased Sensitivity of Surface Plasmon Resonance of Gold Nanoshells Compared to That of Gold Solid Colloids in Response to Environmental Changes. Anal. Chem. 74, 5297–5305 (2002). 12403584 10.1021/ac0258352

[b41] Wang S. *et al.* Collective fluorescence enhancement in nanoparticle clusters. Nat. Commun. 2, 364 (2011) (10.1038/ncomms1357). 21694712

[b42] Shang L. *et al.* Effect of Protein Adsorption on the Fluorescence of Ultrasmall Gold Nanoclusters. Small 8, 661–665 (2012). 22213653 10.1002/smll.201101353

[b43] Ramesh G. V. & Radhakrishnan T. P. A Universal Sensor for Mercury (Hg, HgI, HgII) Based on Silver Nanoparticle-Embedded Polymer Thin Film. ACS Appl. Mater. Interfaces 3, 988–994 (2011). 21395242 10.1021/am200023w

[b44] Lou T., Chen Z., Wang Y. & Chen L. Blue-to-Red Colorimetric Sensing Strategy for Hg^2+^ and Ag^+^ via Redox-Regulated Surface Chemistry of Gold Nanoparticles. ACS Appl. Mater. Interfaces 3, 1568–1573 (2011). 21469714 10.1021/am200130e

[b45] Pu F., Huang Z., Ren J. & Qu X. DNA/Ligand/Ion-Based Ensemble for Fluorescence Turn on Detection of Cysteine and Histidine with Tunable Dynamic Range. Anal. Chem. 82, 8211–8216 (2010). 20806886 10.1021/ac101647k

[b46] Kalluri J. R. *et al.* Use of gold nanoparticles in a simple colorimetric and ultrasensitive dynamic light scattering assay: Selective detection of arsenic in groundwater. Angew. Chem. *In. Ed*. 48, 9668–9671 (2009). 19937875 10.1002/anie.200903958

[b47] Kim Y., Johnson R. C. & Hupp J. T. Gold nanoparticle-based sensing of “spectroscopically silent” heavy metal ions. Nano Lett. 1, 165–167 (2001).

[b48] Elghanian R., Storhoff J. J., Mucic R. C., Letsinger R. L. & Mirkin C. A. Selective Colorimetric Detection of Polynucleotides Based on the Distance-Dependent Optical Properties of Gold Nanoparticles. Science 277, 1078–1081 (1997). 9262471 10.1126/science.277.5329.1078

[b49] Li H. & Rothberg L. Colorimetric detection of DNA sequences based on electrostatic interactions with unmodified gold nanoparticles. PNAS 101, 14036–14039 (2004). 15381774 10.1073/pnas.0406115101 PMC521116

[b50] Lee J. S., Han M. S. & Mirkin C. A. Colorimetric detection of mercuric ion (Hg^2+^) in aqueous media using DNA-functionalized gold nanoparticles. Angew. Chem. Int. Ed. 46, 4093–4096 (2007). 10.1002/anie.200700269 17461429

[b51] Rex M., Hernandez F. E. & Campiglia A. D. Pushing the Limits of Mercury Sensors with Gold Nanorods. Anal. Chem. 78, 445–451 (2006). 16408926 10.1021/ac051166r

[b52] Hu D., Sheng Z., Gong P., Zhang P. & Cai L. Highly selective fluorescent sensors for Hg^2+^ based on bovine serum albumin-capped gold nanoclusters. Analyst 135, 1411–1416 (2010). 20419194 10.1039/c000589d

[b53] Park K. S., Kim M. I., Woo M. A. & Park H. G. A label-free method for detecting biological thiols based on blocking of Hg^2+^-quenching of fluorescent gold nanoclusters. Biosens. Bioelectron. 45, 65–69 (2013). 23454739 10.1016/j.bios.2013.01.047

[b54] Liu Y. & Huang C. Z. Real-Time Dark-Field Scattering Microscopic Monitoring of the *in Situ* Growth of Single Ag^@^Hg Nanoalloys. ACS nano 12, 11026–11034 (2013). 24279755 10.1021/nn404694e

[b55] Mertens S. F. L. *et al.* Au@Hg Nanoalloy Formation Through Direct Amalgamation: Structural, Spectroscopic, and Computational Evidence for Slow Nanoscale Diffusion. Adv. Funct. Mater. 21, 3259–3267 (2011).

[b56] Katok K. V. *et al.* Hyperstoichiometric Interaction Between Silver and Mercury at the Nanoscale. Angew. Chem. Int. Ed. 51, 2632–2635 (2012). 10.1002/anie.201106776 PMC3380547 22307977

