# Supplementary material for: Real-time selective visual monitoring of Hg2+ detection at ppt level: An approach to lighting electrospun nanofibers using gold nanoclusters
Source: Sci Rep. 2015 May 28;5:10403. doi: 10.1038/srep10403 (PMC4446990; doi:10.1038/srep10403)
Supplement: Supplementary Information [file srep10403-s1.doc]

**Supporting Information**

**Real-time selective visual monitoring of Hg^2+^ detection at ppt level: An approach to lighting electrospun nanofibers using gold nanoclusters**

*Anitha Senthamizhan,^*,†^ Asli Celebioglu, ^†,‡^ Tamer Uyar ^*,†, ‡^*

^†^UNAM-National Nanotechnology Research Center, Bilkent University, Ankara, 06800, Turkey

^‡^ Institute of Materials Science & Nanotechnology, Bilkent University, Ankara, 06800, Turkey


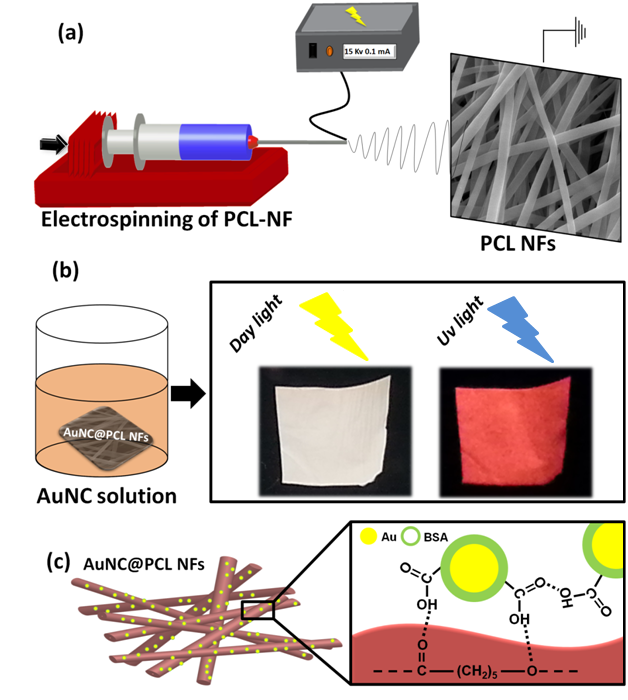


**Figure S1: Schematic representation of the experimental procedure for gold nanoclusters decorated PCL nanofibers (AuNC*PCL-NF). (a) Electrospinning method for producing PCL nanofibers (b) Dip coating method for decorating gold nanoclusters on the PCL nanofiber surface (c) Formation mechanism.**

**Figure S2: DIC image of the (a) PCL nanofibers and (b) AuNC*PCL-NF. It is clearly shown that the diameter is slightly increased after decoration of gold nanoclusters.**

**
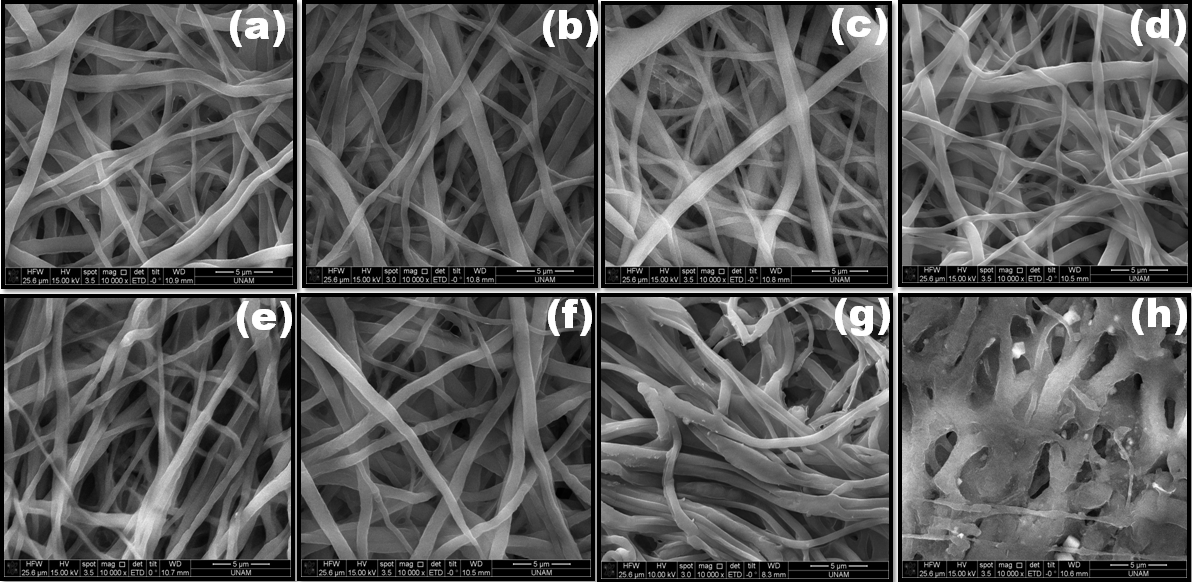
**

**Figure S3: SEM image of the AuNC*PCL-NF with different coating time (a) 10 minutes (b) 30 minutes (c) 1 hour (d) 2 hours (e) 3 hours (f) 6 hours (g) 12 hours (h) 24 hours. Images were taken after washing the membrane with water about 30 minutes.**

**
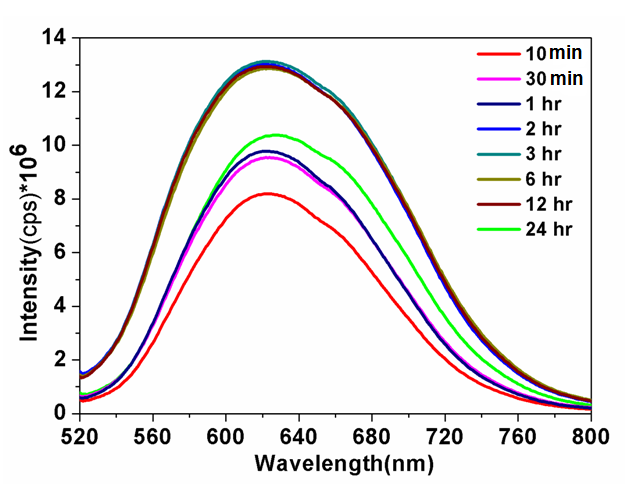
**

**Figure S4: Fluorescence emission spectra of AuNC*PCL-NF with different coating time.**

**Figure S5: CLSM image of the 24 hours AuNC coated PCL nanofibers. The formed patches of the gold nanoclusters on the nanofiber surface enhances the fluorescence of resultant nanofibers meanwhile it decreased the chance to see the nanofiber morphology.**

**
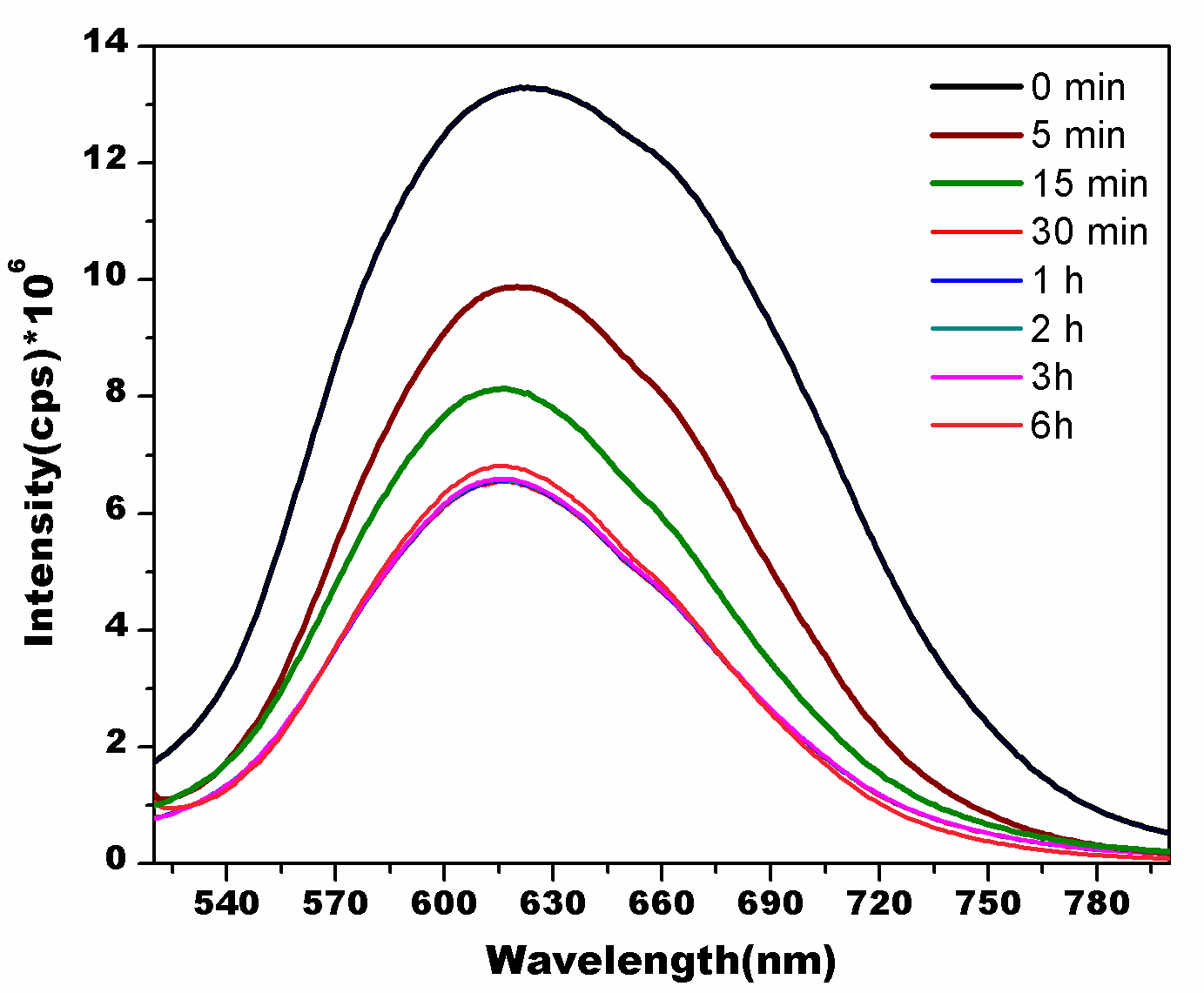
**

**Figure S6: Effect of ligand removal on the emission features of AuNC*PCL-NF. It showing that the excess ligand removed AuNC*PCL-NF has narrower emission profile than unremoved one.**

**Figure S7: CLSM images of AuNC*PCL-NF imaged at various wavelengths from 505-794 nm. Excitation used was 488 nm.**

**Figure S8: Fluorescence images of the AuNC*PCL-NF exposed at typical atmosphere. (a-b) after one month (c) 2 months (d) 4 months and their corresponding emission spectra (e).**

**Figure S9: Photograph of AuNC*PCL-NF treated at different pH values and their relative fluorescence intensity. The observed result confirmed their stability over wide range of pH.**

**
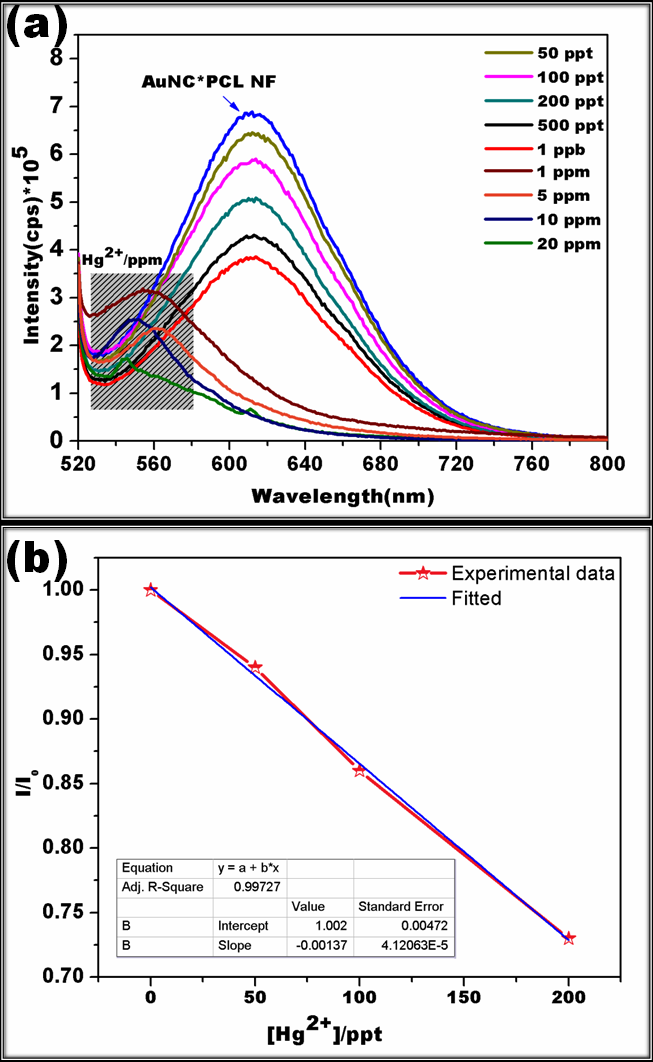
**

**Figure S10: (a) Fluorescence spectra of AuNC*PCL-NF as a function of Hg^2+^ concentration and their (b) variation in relative fluorescence intensity.**

**Figure S11: Variation in the relative fluorescence intensity of AuNC*PCL-NF with different Hg^2+^ concentration in tap water.**

**Figure S12: Effect of salts on the fluorescence nature of AuNC*PCL-NF. Inset shows a photograph of treated AuNC*PCL-NF taken under UV light.**

**Figure S13: Effect of biothiols on the fluorescence nature of AuNC*PCL-NF. Inset shows a photograph of treated AuNC*PCL-NF taken under UV light.**

**Figure S14: Compared sensing performance of as prepared AuNC*PCL-NF towards Cu^2+^. Fluorescence images of the unwashed AuNC*PCL-NF before (a) and after (b) Cu^2+^ exposure at 10 ppm.**

**Figure S15: Real time monitoring of sensing performance. Time dependent CLSM images of AuNC*PCL-NF in the presence of H_2_O (3 µL).**

**Figure S16: Real time monitoring of AuNC*PCL-NF in the presence of H_2_O (6 µL).**

**
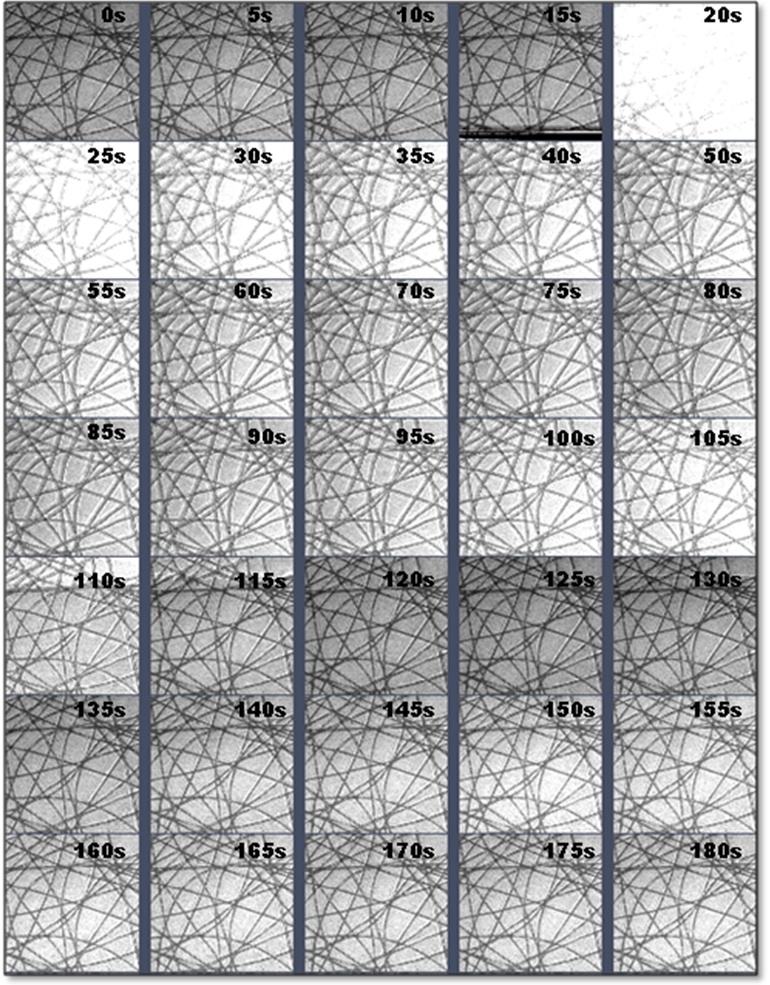
**

**Figure S17: Real time monitoring of AuNC*PCL-NF in the presence of Hg^2+^. DIC images showing that the nanofiber morphology didn’t change after introduction of Hg^2+^.**

**Figure S18: Real time monitoring of AuNC*PCL-NF in the presence of
10 ppm Cu^2+^.**

**
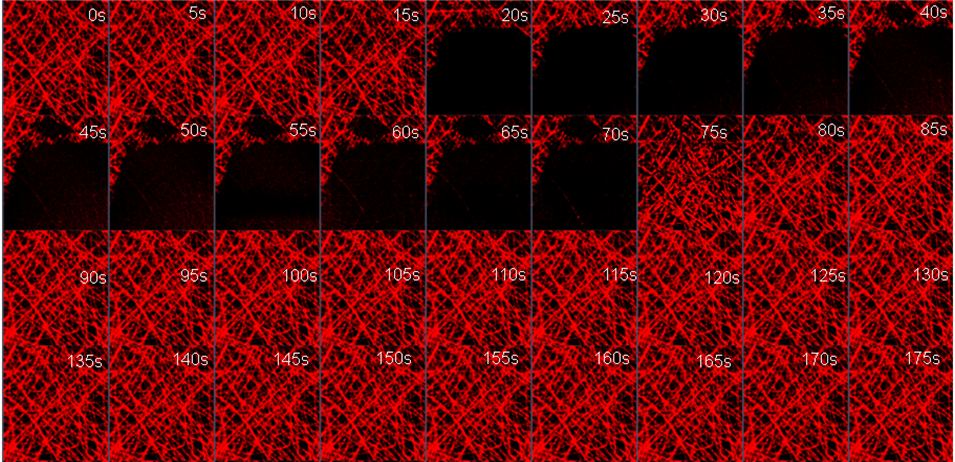
**

**Figure S19: Real time monitoring of AuNC*PCL-NF in the presence of
10 ppm Pb^2+^.**

**Figure S20: Real time monitoring of AuNC*PCL-NF in the presence of
10 ppm Zn^2+^.**

**
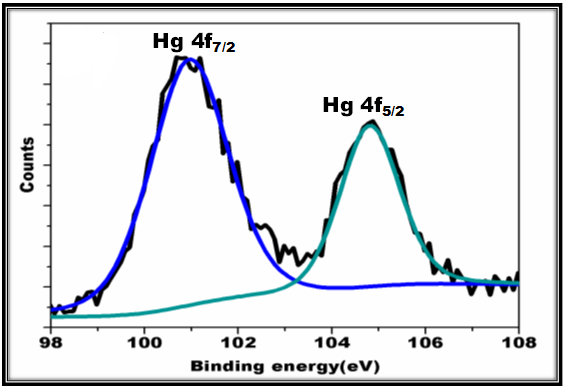
**

**Figure S21: XPS spectra of Hg 4f region of 1 ppm Hg^2+^ treated AuNC*PCL-NF.**
